# Solving the general 3-D safety factor by combining Sarma’s idea with the assumption of normal stress distribution over the slip surface

**DOI:** 10.1371/journal.pone.0287998

**Published:** 2023-06-29

**Authors:** Linghui Wang, Kunlin Lu

**Affiliations:** School of Civil Engineering, Hefei University of Technology, Hefei, China; NUST: National University of Sciences and Technology, PAKISTAN

## Abstract

This study proposes a method for determining 3-D limit equilibrium solutions. The method, inspired by Sarma, introduces the horizontal seismic coefficient as a slope failure parameter and implements a modification of the normal stress over the slip surface. Four equilibrium equations are used to solve the problem without compromising the accuracy of the calculations: three force equilibrium equations in the x, y, and z directions and a moment equilibrium equation in the vertical (z) direction. The reliable factor of safety can be determined by calculating the minimum value of the horizontal seismic coefficient. Furthermore, we analyzed several typical examples of symmetric and asymmetric slopes, finding good consistency with the existing literature. This consistency indicates the reliability of the factor of safety we obtained. The proposed method is favored due to its straightforward principle, convenient operation, fast convergence, and ease of programming.

## 1. Introduction

Slope stability is a classical problem in soil mechanics, and it is ubiquitous in the field of geotechnical engineering. Existing methods include the limit analysis method [1,2], the limit equilibrium method [3], the strength reduction method [4], and the variational analysis [5]. Most of which exist because there is no established unifying method for slope stability. Two-dimensional (2-D) slope stability analysis methods are most commonly used among engineers due to their simplicity. These 2-D methods are based on simplified assumptions, and in this way, they reduce the dimension of the problem from 3-D to 2-D. For a known slip surface, 2-D methods are based on the limit equilibrium theory that usually employs the method of slices, including Bishop[6], Morgenstern and Price [7], Spencer[8], Janbu[9], Sarma [10], Fredlund and Krahn [11], and so on.

The complexity of slip surfaces in 3D has rendered 2D methods incapable of yielding precise results. Accordingly, tentative development of 3D methods has taken place, including those by Xing [12], Hungr et al. [13], and Huang and Tsai [14]. For a given slip surface, the factor of safety is predominantly calculated under partial equilibrium conditions using non-rigorous 3D methods, such as those developed by Lam and Fredlund [15], Huang et al. [16], and Chen and Zhu [17]. Efforts have been made by Hovland [18], Hungr et al. [13], and Boutrup and Lovell [19] to extend 2D models, originally developed by Fellenius [3], Bishop [6], and Janbu [9], to their 3D counterparts. However, such extensions are associated with assumptions about inter-column forces, which may lead to divergent results in iterative calculations.

The safety factor can be obtained if the distribution of the normal stress can be determined in the slope stability analysis. The assumed distribution of the normal stress acting on the slip surface is proposed by Bell [20], which is a function with two parameters to be determined. Zhu and Lee [21] assume that the distribution of the normal stress acting on the slip surface is composed of an initial function and a modified function, the latter of which was assumed to be a linear interpolation function with two parameters to be determined. This method does not need to assume the inter-column forces, thereby compensating for flaws in the integration of calculations in conventional methods. Zhu et al. [22] extend the assumption of the normal stress to the 3D limit equilibrium, only considering the vertical force equilibrium and the moment equilibrium by rotating axis for symmetric slopes. Moreover, they establish a 3-D calculation method that satisfies different equilibrium conditions for the slope stability based on this concept [23–25]. Zhu and Qian [26] suggest that the modified normal stress distribution along the sliding surface should be non-negative. The methods proposed by Zhu and Qian [26] and Lu and Zhu [27] need to solve the strict limit equilibrium solution from a nonlinear system that contains six variables.

Our team presents a simplified method for calculating the factor of safety of a slope with a three-dimensional (3-D) slip surface. This method satisfies all six equilibrium conditions for the 3-D sliding body. By incorporating Sarma’s concept, we introduce a clever trick that employs the horizontal seismic force coefficient to linearize the equations [28]. Through this, we obtain a rigorous limit equilibrium solution for 3-D slope stability. However, there are cases where negative normal stress may occur over the surface edge, necessitating modifications. This process is lengthy and complicated, it is difficult for engineers to control and apply. Therefore, we propose utilizing four equilibrium equations to address the problem without compromising the accuracy of the calculation. It involves disregarding two minor equilibrium conditions, the moments about the x- and z-axis, rendering it easily applicable to engineering calculations. The efficacy of the proposed method has been evaluated by applying it to a selection of both symmetric and asymmetric classic three-dimensional slope examples. The results have demonstrated that the method is both quick and reliable.

## 2. Methodology

### 2.1 Basic assumptions

**Fig 1:**
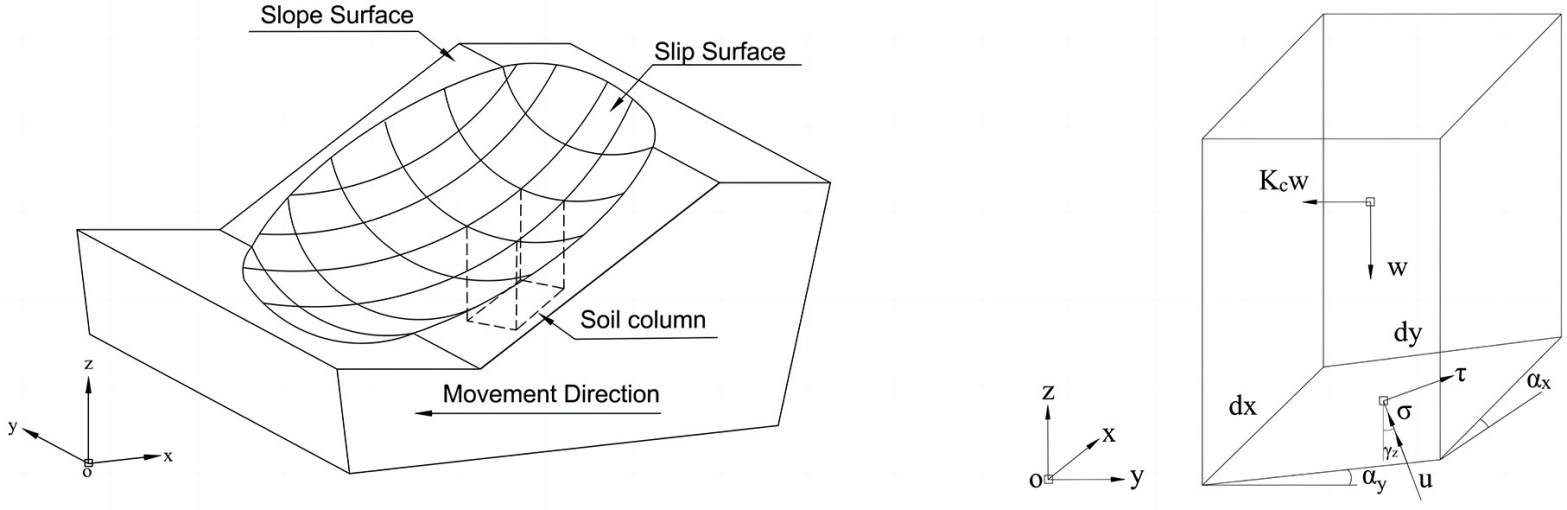
(a). 3-D collapsed slope and (b) forces acting on a column.

Fig 1(A) proposed a 3-D collapsed slope, it is assumed that the direction of the slip surface is opposite to the x-axis, and the sliding mass is discretized into *n* columns. The slope and slip surface are described by functions *s*(*x*,*y*) and *g*(*x*,*y*), respectively. There are three forces acting on a typical discretized column, including: (i) the weight of the column, *w*(*x*,*y*), which numerically equal γ(x,y)[s(x,y)−g(x,y)], where *γ*(*x*,*y*) is the unit soil weight, *τ*(*x*,*y*) is the shear stress at the base, (ii) the normal stress at the base, *σ*(*x*,*y*), and (iii) the horizontal seismic coefficient, *K*_*c*_, as shown in Fig 1(B). The discretization widths of the column in the x- and y-directions are defined as d*x* and d*y*, respectively. *α*_*x*_ and *α*_*y*_ are the horizontal inclination angles of the slip surface along the x- and y-directions, respectively. According to the geometric of the column information, we have:

$$s_{x}^{\prime }=\tan\alpha_{x}$$ (1)

$$s_{y}^{\prime }=\tan\alpha_{y}$$ (2)

$$\cos\gamma_{z}=\frac{1}{\sqrt{{s^{\prime }}_{x}^{2}+{s^{\prime }}_{y}^{2}+1}}$$ (3)

$$\Delta =\sqrt{{s^{\prime }}_{x}^{2}+{s^{\prime }}_{y}^{2}+1}$$ (4)

where *γ*_*z*_ is the angle between the normal of the slip surface and the z-axis. $s_{x}^{'}$ and $s_{y}^{'}$ are the tangent values that the angle between the column base and x- and y-axis directions. In the three-dimensional slope stability analysis, it is necessary to analyze and calculate the directions of both normal stress (*σ*) and shear stress (*τ*). The cosine direction of the normal stress over the slip surface can be represented by $\left(n_{\sigma }^{x},n_{\sigma }^{y},n_{\sigma }^{z}\right)$, where:

$$n_{\sigma }^{x}=-\frac{s_{x}^{\prime }}{\Delta }$$ (5)

$$n_{\sigma }^{y}=-\frac{s_{y}^{\prime }}{\Delta }$$ (6)

$$n_{\sigma }^{x}=\frac{1}{\Delta }$$ (7)

The direction of the potential sliding mass is generally parallel to the x-axis, while the direction of shear stress is perpendicular to the direction of normal stress. Therefore, the cosines of the shear stress *τ* can be written as $\left(n_{\tau }^{x},n_{\tau }^{y},n_{\tau }^{z}\right)$, where:

$$n_{\tau }^{x}=\frac{1}{\Delta^{\prime }}$$ (8)

$$n_{\tau }^{y}=0$$ (9)

$$n_{\tau }^{z}=\frac{s_{x}^{\prime }}{\Delta^{\prime }}$$ (10)

Where $\Delta =\sqrt{1+s_{x}^{\prime }{}^{2}}$.

**Fig 2:**
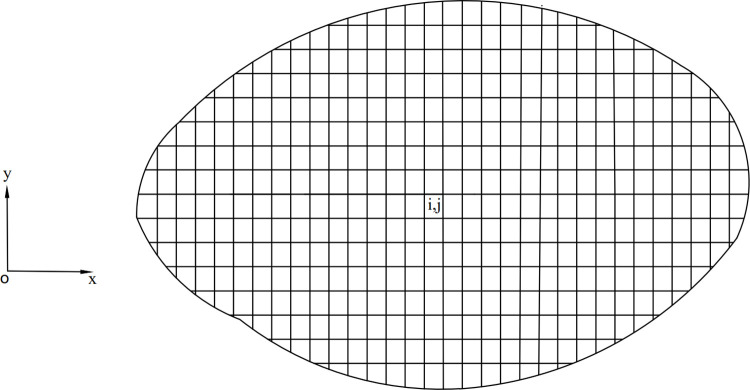
Projection of 3-D failure mass in the x-y plane.

As shown in Fig 2, there are many rectangles when the slip surface of a small column is projected to the x-y plane. Each rectangle has a base area of d*A*, which can be expressed as:

dA=Δdxdy (11)

The initial normal stress over the slip surface is obtained by applying Hovland method [18]:

$$\sigma_{0}=\frac{w\cdot \cos\gamma_{z}}{\Delta }=\frac{w}{{s^{\prime }}_{x}^{2}+{s^{\prime }}_{y}^{2}+1}$$ (12)

where *w* is the weight of the column.

In this paper, the proposed normal stress distribution over the slip surface is described by Eq (13), while the modification function is shown in Eq (14), both of which are from Zhu and Qian [26]:

$$\sigma (x,y)=\sigma_{0}(x,y)\xi (x,y)$$ (13)

$$\xi (x,y)=\xi_{0}(x,y)+\lambda_{1}\cdot \xi_{1}(x,y)+\lambda_{2}\cdot \xi_{2}(x,y)+\lambda_{3}\cdot \xi_{3}(x,y)$$ (14)

where *σ*(*x*,*y*) is the total normal stress distribution when the sliding mass is in the limit equilibrium state. Based on the limit equilibrium methods, the proposed method considers the sliding mass as a rigid mass in the limit equilibrium state, and it satisfies four equations of limit equilibrium according to the assumption of normal stress. Herein, the modification function is defined as

$$\xi (x,y)=\lambda_{1}+\lambda_{2}\cdot x+\lambda_{3}\cdot y$$ (15)

where *λ*_1_, *λ*_2_ and *λ*_3_ are unknown parameters.

### 2.2 The limit condition

To ensure the consistency with the limit equilibrium approach and the validity of the Mohr-Coulomb criterion, the shear strength, *τ*, is defined as:

$$\tau =\frac{(\sigma -u)\tan\varphi^{\prime }+c^{\prime }}{F_{s}}=\frac{\sigma \psi +c_{u}}{F_{s}}$$ (16)

where *c*′ and *ψ* are the soil cohesion and the friction coefficient, respectively; *ψ* = tan*φ*′, where *φ* is the effective angle of internal friction; *c*_*u*_ = *c*′−*uψ*, where *u* is the pore water pressure and *F*_*s*_ is the slope safety factor.

### 2.3 Equilibrium equations of the failure mass

In a three-dimensional sliding body, the proposed approach satisfies the force equilibrium in all three directions and the moment equilibrium in the vertical direction:

$$\iint (\sigma n_{\sigma }^{x}+\tau n_{\tau }^{x})\Delta dxdy-\iint K_{c}wdxdy=0$$ (17A)

$$\iint (\sigma n_{\sigma }^{y}+\tau n_{\tau }^{y})\Delta dxdy=0$$ (17B)

$$\iint (\sigma n_{\sigma }^{z}+\tau n_{\tau }^{z})\Delta dxdy-\iint wdxdy=0$$ (17C)

$$\iint (\sigma n_{\sigma }^{z}+\tau n_{\tau }^{z})x\Delta dxdy-\iint (\sigma n_{\sigma }^{x}+\tau n_{\tau }^{x})s\Delta dxdy-\iint wxdxdy+\iint K_{C}wz_{c}dxdy=0$$ (17D)

Substituting Eq (16) into Eqs (17A)–(17D) respectively yields:

$$\iint \sigma (n_{\sigma }^{x}\Delta +\frac{1}{F_{s}}\psi n_{\tau }^{x}\Delta )dxdy+\frac{1}{F_{s}}\iint c_{u}n_{\tau }^{x}\Delta dxdy=\iint K_{c}wdxdy$$ (18A)

$$\iint \sigma (n_{\sigma }^{y}\Delta +\frac{1}{F_{s}}\psi n_{\tau }^{y}\Delta )dxdy+\frac{1}{F_{s}}\iint c_{u}n_{\tau }^{y}\Delta dxdy=0$$ (18B)

$$\iint \sigma (n_{\sigma }^{z}\Delta +\frac{1}{F_{s}}\psi n_{\tau }^{z}\Delta )dxdy+\frac{1}{F_{s}}\iint c_{u}n_{\tau }^{z}\Delta dxdy=wdxdy$$ (18C)

$$\begin{array}{l} \iint \sigma [(n_{\sigma }^{x}s-n_{\sigma }^{z}x)\Delta +\frac{1}{F_{s}}(n_{\tau }^{x}s-n_{\tau }^{z}x)\psi \Delta ]dxdy+\frac{1}{F_{s}}\iint c_{u}(n_{\tau }^{x}s-n_{\tau }^{z}x)\Delta dxdy\\ =-\iint wxdxdy+\iint K_{c}wz_{c}dxdy \end{array}$$ (18D)

Eqs (18A)–(18D) can be abbreviated as:

$$\iint \sigma (f_{i1}+\frac{1}{F_{s}}f_{i2})dxdy+\frac{1}{F_{s}}\iint f_{i3}dxdy=\iint f_{i4}dxdy+K_{c}\iint d_{i}dxdy(i=1,2,3,4)$$ (19)

### 2.4 Numerical solution for the factor of safety

*K*_*c*_ is set as a known physical parameter to discuss its effect on the slope stability analysis. The proposed method determines the factor of safety, *F*_*s*_ for a slope in a different way than conventional methods, because it considers two to-be-known parameters, namely *K*_*c*_ and *F*_*s*_. Usually, *F*_*s*_ is assumed to be 1, while *K*_*c*_ required to produce this *F*_*s*_ is solved as an unknown. The linear equations of the four variables can be obtained by substituting Eq (13) into Eq (19), yielding:

$$\begin{array}{l} \sum_{j=1}^{3}\lambda_{j}\iint \sigma_{0}\xi_{j}(f_{i1}+\frac{1}{F_{s}}f_{i2})dxdy-K_{c}\iint d_{i}dxdy=\iint f_{i4}dxdy-\frac{1}{F_{s}}\iint f_{i3}dxdy\\ -\iint \sigma_{0}\xi_{0}f_{i1}dxdy(i=1,2,3,4) \end{array}$$ (20)

Eq (20) can be rewritten in the matrix form:

$$\left[A]{\left[\lambda \right]}^{T}=[B\right]$$ (21)

When *i* = 1, 2, 3 and *j* = 1, 2, 3, we have:

$$A_{ij}=\iint \sigma_{0}\xi_{j}(f_{i1}+\frac{1}{F_{s}}f_{i2})dxdy$$ (21A)

When *i* = 1,2,3,4 and *j* = 4, we have:

$$A_{ij}=-\iint d_{i}dxdy$$ (21B)

$$B_{i}=\iint f_{i4}dxdy-\frac{1}{F_{s}}\iint f_{i3}dxdy-\iint \sigma_{0}\xi_{0}f_{i1}dxdy(i=1,2,3,4)$$ (21C)

The matrix can quickly solve the four equations, and their formulas are listed in Table 1.

**Table 1:** Calculation parameters for three-dimensional slope stability analysis.

Formulas				
$\begin{array}{l} f_{11}=n_{\sigma }^{x}\cdot \Delta \\ f_{21}=n_{\sigma }^{y}\cdot \Delta \\ f_{31}=n_{\sigma }^{z}\cdot \Delta \\ f_{41}=(n_{\sigma }^{x}\cdot s-n_{\sigma }^{z}\cdot x)\cdot \Delta \end{array}$	$\begin{array}{l} f_{12}=\psi n_{\tau }^{x}\Delta \\ f_{22}=\psi n_{\tau }^{y}\Delta \\ f_{32}=\psi n_{\tau }^{z}\Delta \\ f_{42}=\psi (n_{\tau }^{x}s-n_{\tau }^{z}x)\Delta \end{array}$	$\begin{array}{l} f_{13}=c_{u}n_{\tau }^{x}\Delta \\ f_{23}=c_{u}n_{\tau }^{y}\Delta \\ f_{33}=c_{u}n_{\tau }^{z}\Delta \\ f_{43}=c_{u}(n_{\tau }^{x}s-n_{\tau }^{z}x)\Delta \end{array}$	$\begin{array}{l} f_{14}=0\\ f_{24}=0\\ f_{34}=w\\ f_{4}{}_{4}=-wx \end{array}$	$\begin{array}{l} d_{1}=w\\ d_{2}=0\\ d_{3}=0\\ d_{4}=wz_{c} \end{array}$


$$A=[\begin{array}{cccc} A_{11} & A_{12} & A_{13} & A_{14}\\ A_{21} & A_{22} & A_{23} & A_{24}\\ A_{31} & A_{32} & A_{33} & A_{34}\\ A_{41} & A_{42} & A_{43} & A_{44} \end{array}]$$ (22A)

$$\lambda ={\left[\begin{array}{cccc} \lambda_{1} & \lambda_{2} & \lambda_{3} & K_{c} \end{array}\right]}^{T}$$ (22B)

$$B=[\begin{array}{cccc} B_{1} & B_{2} & B_{3} & B_{4} \end{array}]$$ (22C)

$$\lambda =A^{-1}B$$ (22D)

As is indicated in Eq (20), *K*_*c*_ exists in a linear equation system and can, through the matrix, solve Eq (22C).

To calculate *F*_*s*_ for a given slip surface, *K*_*c*_ is needed to implement several iterations by changing the value of *F*_*s*_. Eq (23) is used to search for the minimum *K*_*c*_ and the corresponding *F*_*s*_:

$$F_{s}=F_{s_{1}}+\frac{F_{s_{2}}-F_{s_{1}}}{K_{c_{2}}-K_{c_{1}}}(-K_{c_{1}})$$ (23)

### 2.5. Solution for the safety factor

**Fig 3:**
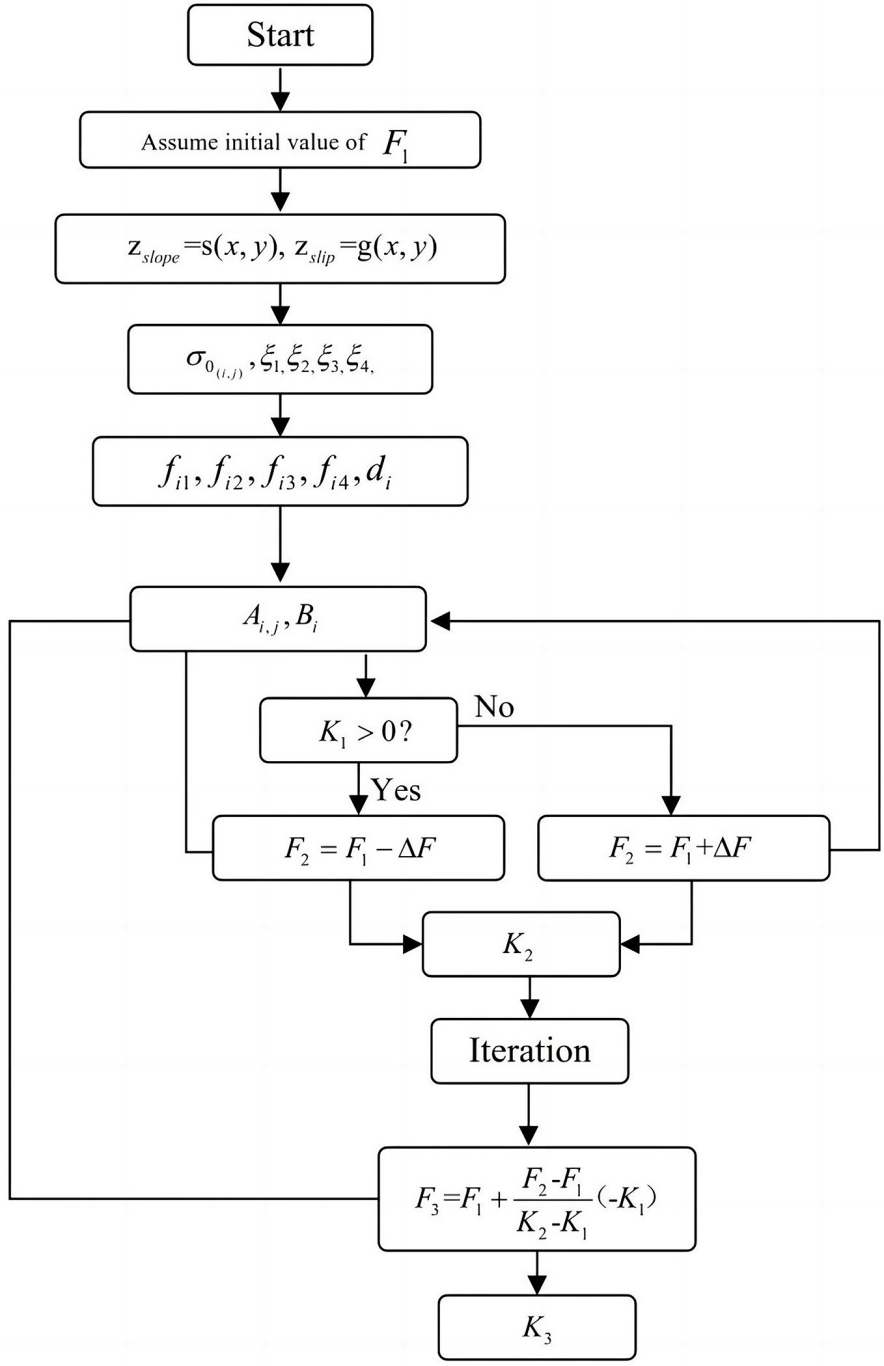
Flowchart showing how F_s_ is determined in the three-dimensional slope stability analysis.

This study aims to find the minimum *K*_*c*_ that can determine *F*_*s*_. Specifically, *F*_*s*_ is negatively correlated with *K*_*c*_; that is, a larger horizontal seismic force is needed to cause the slope sliding for a more stable slope [29,30]. Therefore, *K*_*c*_ would be very small if the slope is in a very stable state. There are several steps to compute *F*_*s*_ using the present framework, as shown in Fig 3:

1. Divide the failure mass into *n* columns.
2. Calculate the assumed initial normal stress over the slip surface *σ*_0_ and its modification *ξ*_*i*_.
3. Calculate the values of *f*_*i*1_, *f*_*i*2_, *f*_*i*3_ and *f*_*i*4_.
4. Assume an initial iteration value of the safety factor *F*_*s*_ as 1, which is expressed as *F*_1_.
5. Compute the value of *K*_1_ and the second value of safety factor *F*_2_.
6. Replace *F*_1_ by *F*_2_ and return to calculate *K*_2_.
7. Calculate the value of *F*_3_ according to Eq (26).
8. Use the value of *F*_3_ to calculate the value of *K*_3_.
9. Calculate the difference Δ*F*_1_ between *F*_3_ and *F*_1_, and the difference Δ*F*_2_, between *F*_2_ and *F*_1_.
10. Replace *F*_2_ by *F*_3_ and *K*_3_ by *K*_2_ when Δ*F*_1_ is smaller than Δ*F*_2_; otherwise, replace *F*_2_ by *F*_1_, *K*_2_ by *K*_1_, *F*_3_ by *F*_1_, and *K*_3_ by *K*_2_.
11. Repeat steps 4 to 10 until |*K*_3_−*K*_2_|<*ε*.

The iteration procedure is to repeatedly change the values of *F*_*s*_ to get different value of *K*_*c*_ until |*K*_3_−*K*_2_|<*ε* that indicates *K*_3_ to be close to zero (Part 2 in Fig 3). Hence, the final value of *K*_*c*_ corresponds to the actual factor of safety of the three-dimensional slope. Due to the linear relationship between *K*_*c*_ and *λ*, only several times of iterations are needed to calculate the actual value of *F*_*s*_.

## 3. Numerical examples and verification

A computer program is developed in MATLAB to illustrate the 3D shape of the failure surface and calculate its safety factor. Four examples are performed to evaluate the feasibility of this program for stability analysis of slopes with ellipsoid and asymmetric slip surfaces.

### 3.1. Example 1: A ellipsoid slip surface with weak layer

**Fig 4:**
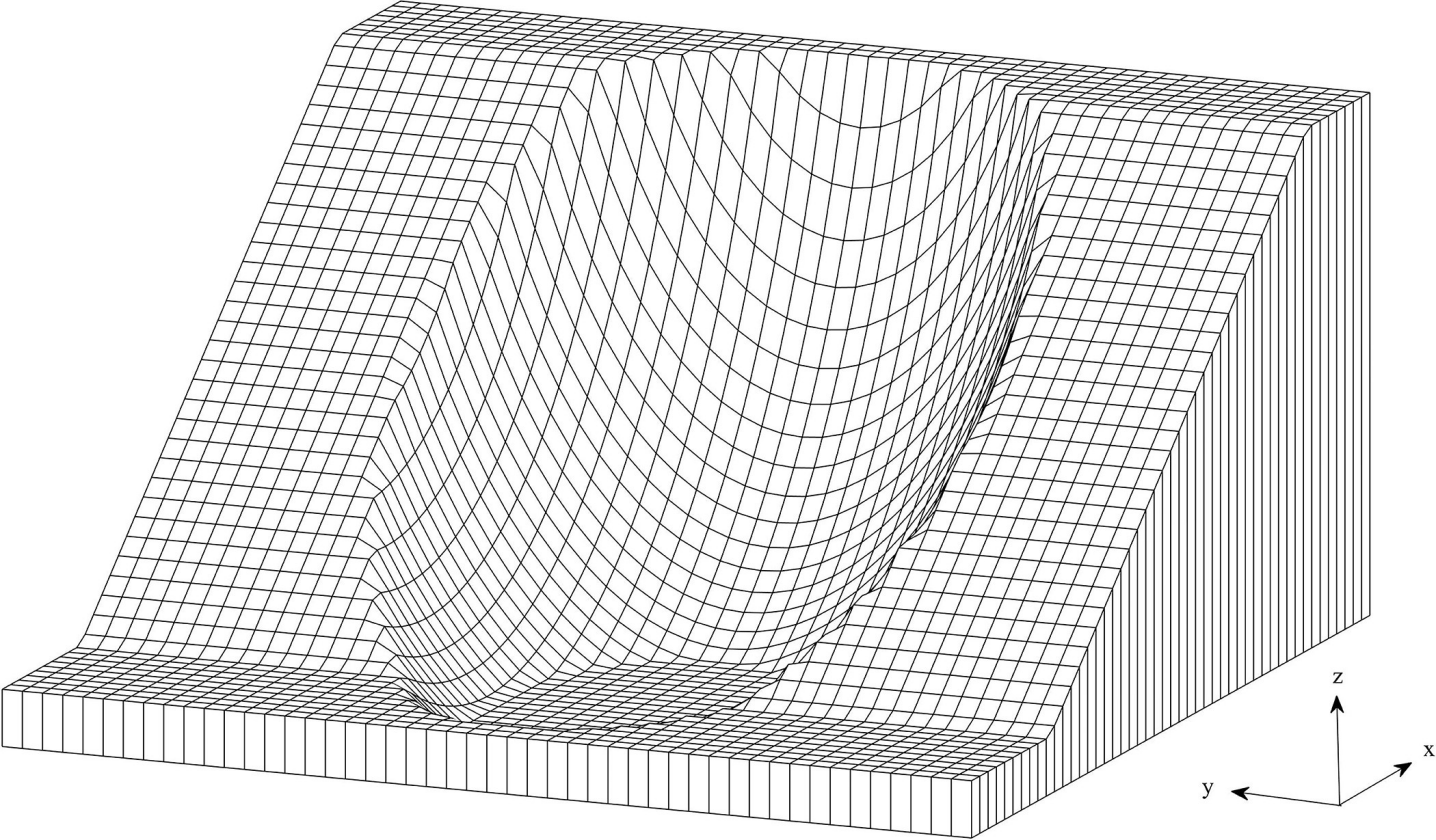
The 3D slope profiles used in Examples 1.

**Fig 5:**
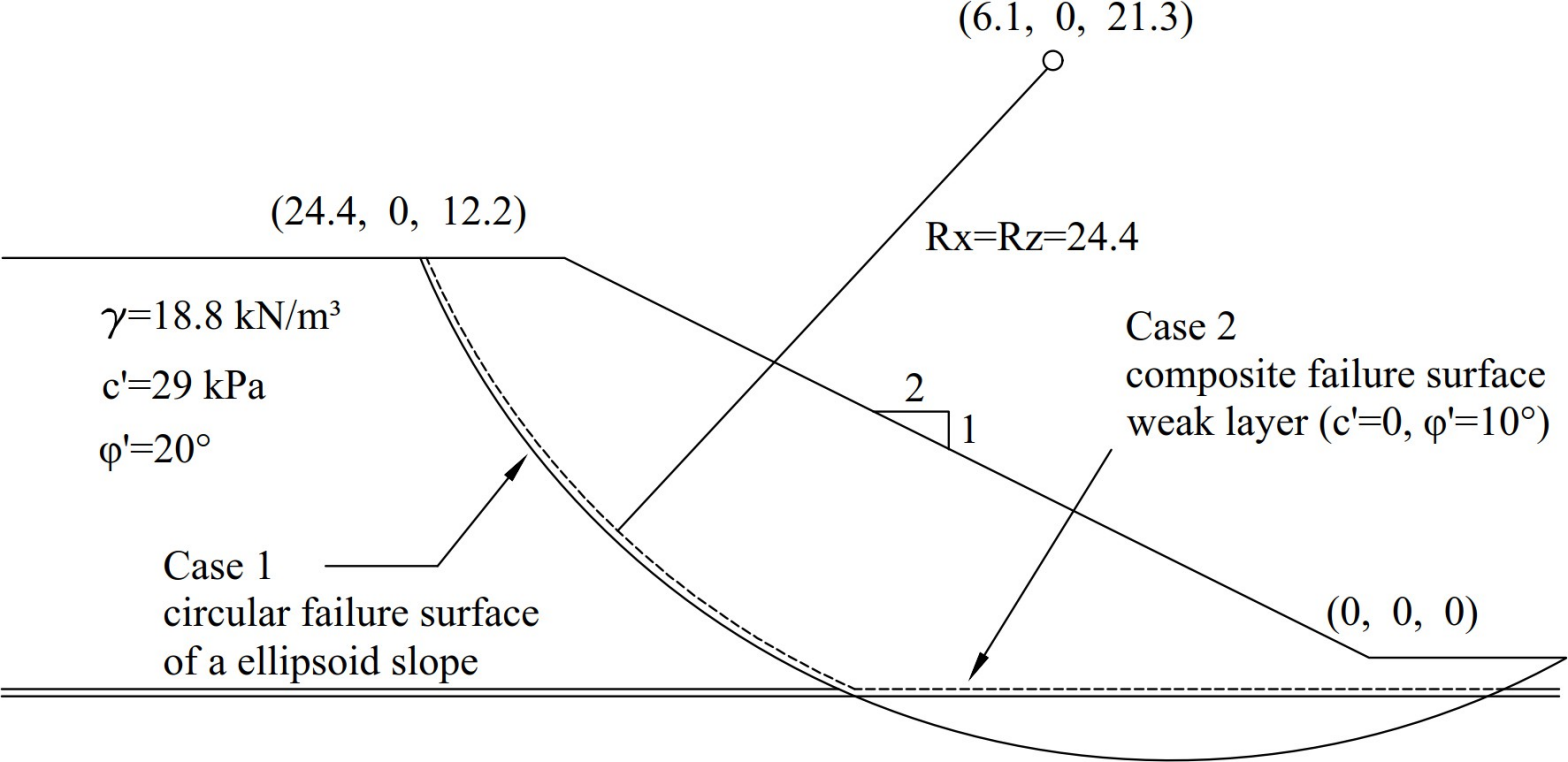
Cross-section and parameters of an ellipsoid slope.

The proposed method is first validated in 2 cases using the 3D example dataset provided by Xing [12]. Case 1 has a failure surface that is assumed to be a symmetric ellipsoid, while Case 2 has a failure surface that is composed of an ellipsoid surface and a weak layer. The 3D slope profile used in Example 1 is presented in Fig 4. They have been studied by many researchers, including Xing[12], Hungr et al.[13], Huang and Tsai [14], Chen and Zhu [17], Zhu and Qian[26], Lu and Zhu[27], Zhu et al. [28] and Chen et al. [31]. The cross-section and parameters are shown in Fig 5, including a slope height *(H)* of 10 m and a slope ratio of 1:2. Soil strength parameters in Case 1 are as follows: cohesion *c*′ = 29 *kPa*, internal angle *φ*′ = 20°, and natural unit gravity *γ* = 18.8 *kN*/*m*^3^. The equation of the slip surface can be expressed as

$$\frac{{\left(x-6.1\right)}^{2}}{24.4^{2}}+\frac{y^{2}}{78.2^{2}}+\frac{{\left(z-21.3\right)}^{2}}{24.4^{2}}=1$$ (24)

As shown in Table 2, the proposed method obtained similar values of *F*_*s*_ on the composite failure surface with those by previous methods for Case 1. The safety factor calculated by the proposed method is 2.142, compared to the 2.122 value given by Xing [12]. Both methods satisfy force equilibrium in three directions and moment equilibrium in the vertical direction, signifying that the set of equilibrium conditions is similar for both. However, the result from our proposed method is larger than that of Xing [12], which can be attributed to the omission of the intercolumn force during our calculation. The difference between the method used in this study and the Rigorous 3-D methods used by Zhu and Qian [26] and Lu and Zhu [27] is.0.75% and 0.05%, respectively, which is essentially negligible. This fact validates the feasibility of the proposed method in solving for the safety factor using four equilibrium equations. The high similarity between the results of the proposed method and Zhu and Qian [28] may stem from the same assumptions used in these two methods. Compared with other methods such as those by Hungr et al. [13], Huang and Tsai [14], Chen and Zhu [17], and Chen et al. [31], the errors are 1.17%, 3.41%, 3.17%, and 2.1%, respectively. These errors fall within an acceptable range, demonstrating that the results obtained by this method are in line with those from previous methods and thus are acceptable.

**Table 2:** Comparisons of calculated safety factors by using different methods.

Methods	Case 1		Case 2	
	Fs	Error	Fs	Error
The proposed method	2.142	-	1.662	-
Xing [12]	2.122	0.93%	1.533	7.76%
Hungr et al. [13]	2.167	1.17%	1.620	2.53%
Huang and Tsai [14]	2.215	3.41%	1.665	0.18%
Chen and Zhu [17]	2.210	3.17%	-	-
Zhu and Qian [26]	2.158	0.75%	1.660	0.12%
Lu and Zhu [27]	2.141	0.05%	1.662	0.00%
Zhu et al. [28]	2.141	0.05%	1.661	0.06%
Chen et al. [31]	2.187	2.10%	1.640	1.32%

Note: The error refers to the difference between other methods and the proposed method.

In Case 2, as shown in Table 2, the safety factor calculated by the proposed method is 1.662. An 7.76% difference exists between the results of the proposed method and those from Xing [12]. This discrepancy could be attributed to the shear force on the two side faces of all columns along the slip direction, which was ignored in our proposed method. Similarly, the results from our method are consistent with those of Lu and Zhu [27]. The discrepancies between our method and the methods of Zhu and Qian [26] and Zhu et al. [28] are 0.12% and 0.06%, respectively. The high similarity in safety factors can be attributed to the assumption employed regarding the normal stress distribution along the slip surface. The difference with Hungr et al. [13], Huang and Tsai [14], Chen and Zhu [17], and Chen et al. [31] may be caused by the following factors: (1) Different number of the discrete columns; (2) Different assumption of inter-column force; (3) Different assumptions of normal stress distribution.

**Fig 6:**
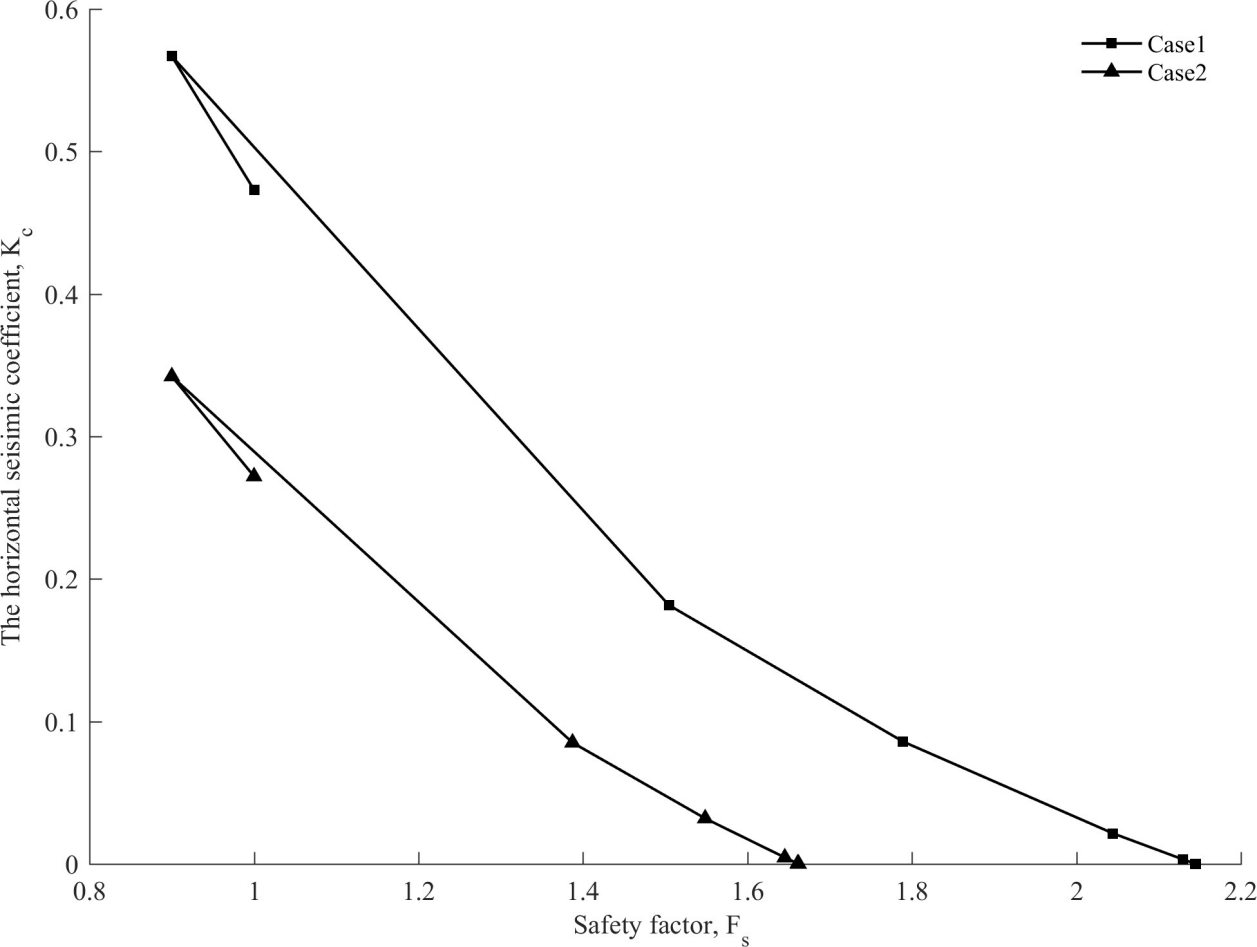
Correlations between *K*_*c*_ and *F*_*s*_ for an ellipsoid surface and a composite surface, respectively.

As shown in Fig 6, the safety factor of the slope increases as the horizontal seismic coefficient decreases in all iteration steps.

**Fig 7:**
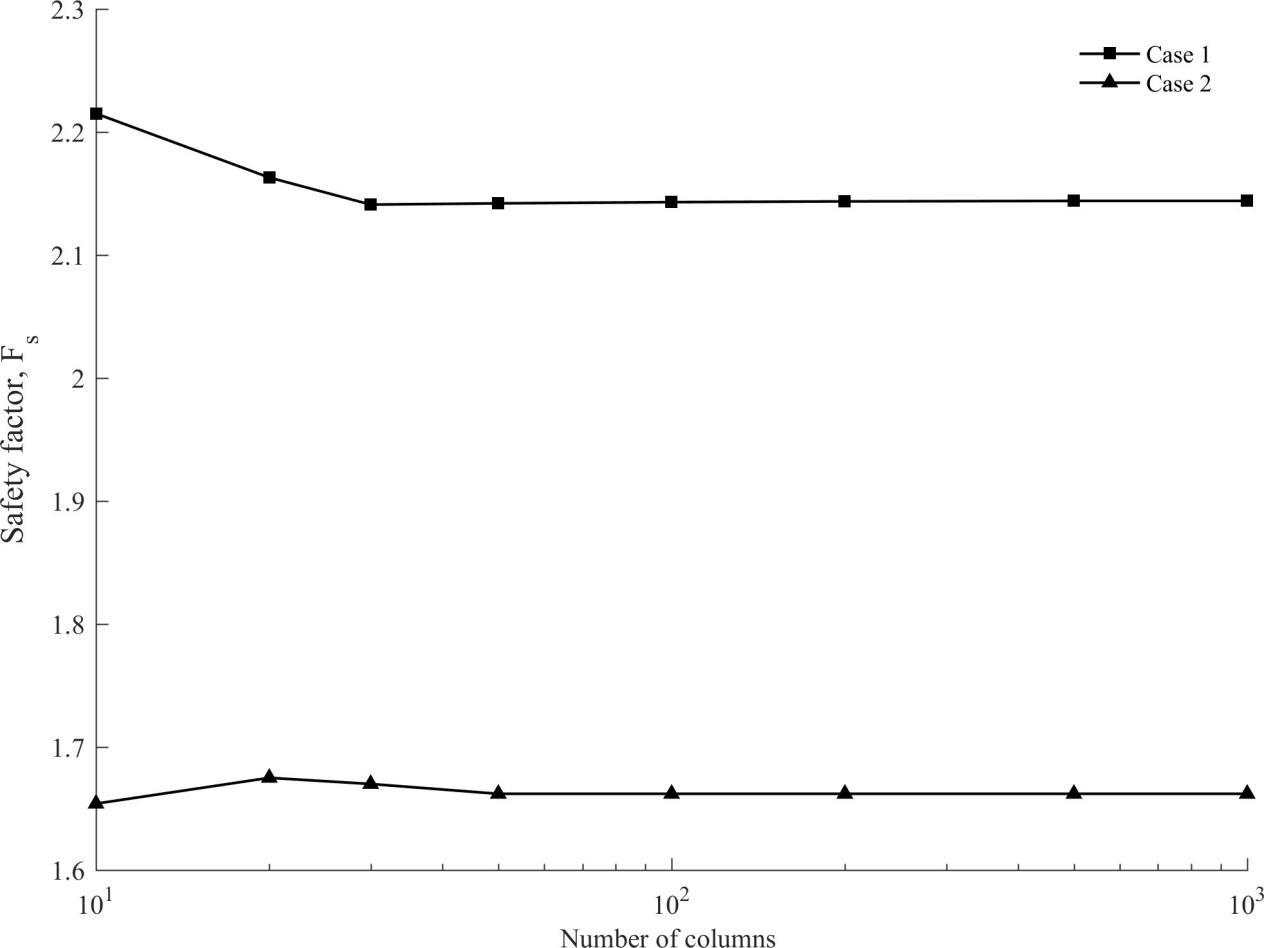
The effect of mesh size on the safety factor.

Various columns were used to discretize the failure mass to examine the effects of the number of columns on solution accuracy. Fig 7 illustrates the grid sensitivity. The safety factor ranges from 2.139 to 2.215 in Case 1, while it varies between 1.654 and 1.675 in Case 2. The safety factor gradually stabilizes as the number of columns increases. Therefore, it can be indicated that the safety factor converged to a certain value as the columns increased.

**Fig 8:**
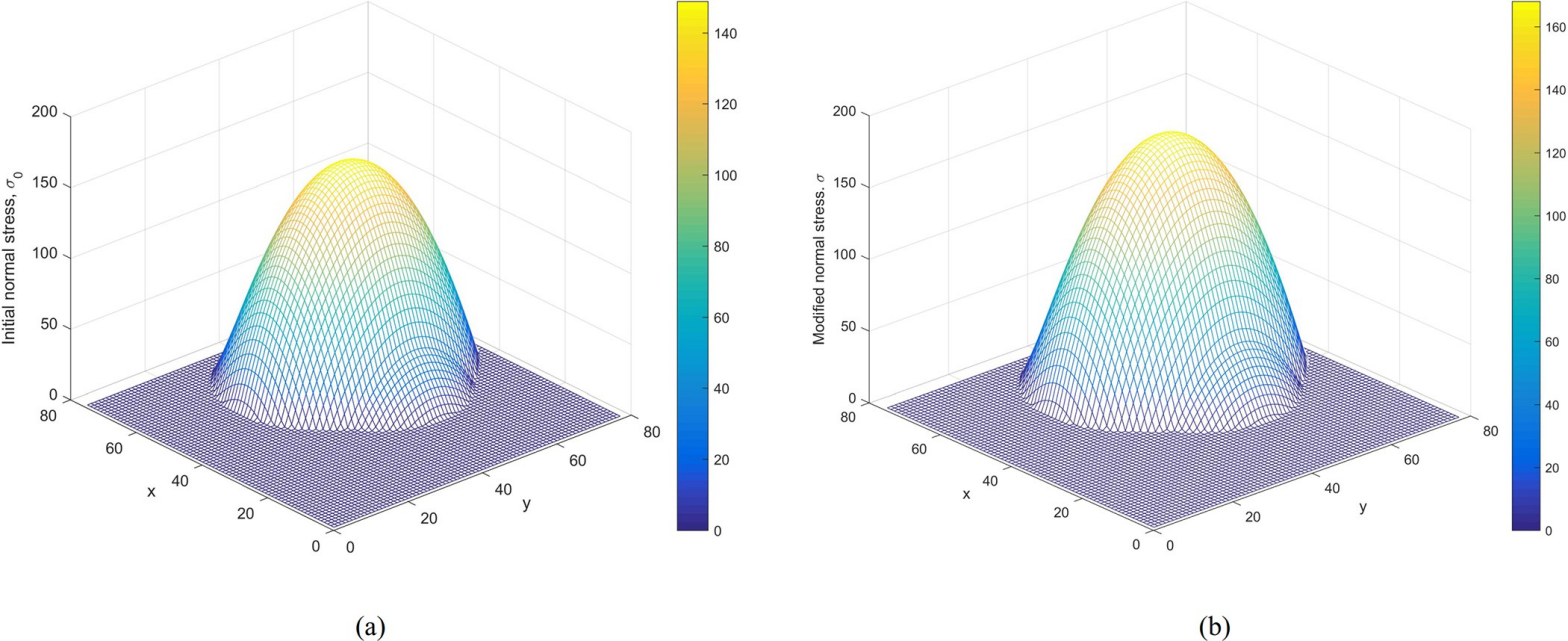
Case 1: (a) The distribution of the initial normal stress over the slip surface. (b) The distribution of the modified normal stress over the slip surface.

**Fig 9:**
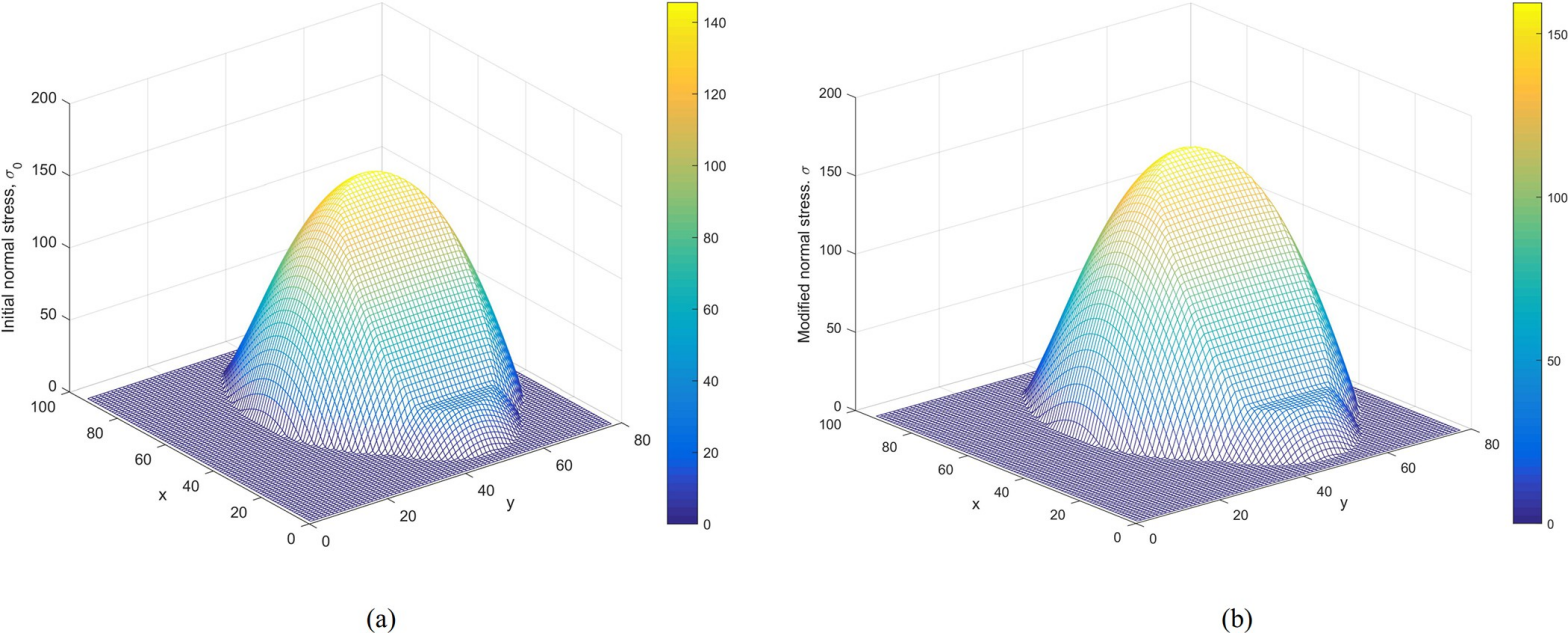
(a) The distribution of initial normal stress over the slip surface. (b) The distribution of modified normal stress over the slip surface. (Case 2).

**Verification of the modified normal stress.** Figs 8 and 9 show the distributions of initial normal stress and the modified normal stress over the slip surface of Case 1 and Case 2, respectively. The modified normal stresses over the slip surface are positive, smooth, and continuous. Therefore, the results are reasonable.

### 3.2. Example 2: A homogenous slope from Lu and Zhu [27]

**Fig 10:**
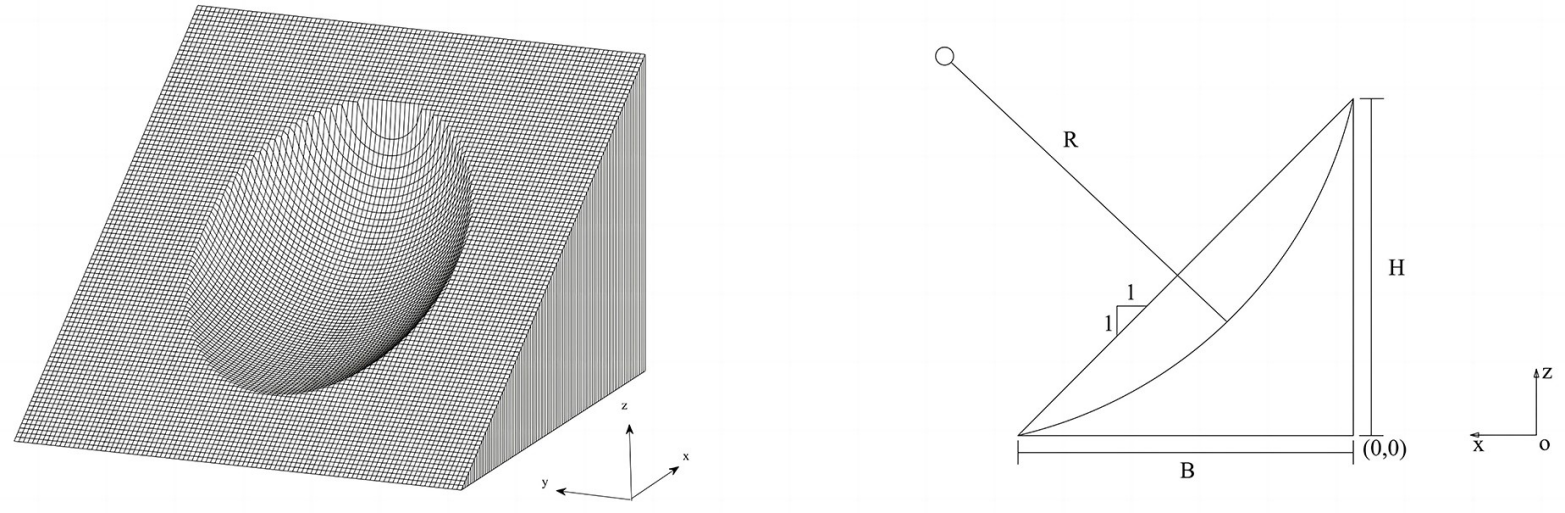
(a) The 3D profile model of example 2. (b) Cross-section and parameters of example 2.

**Fig 11:**
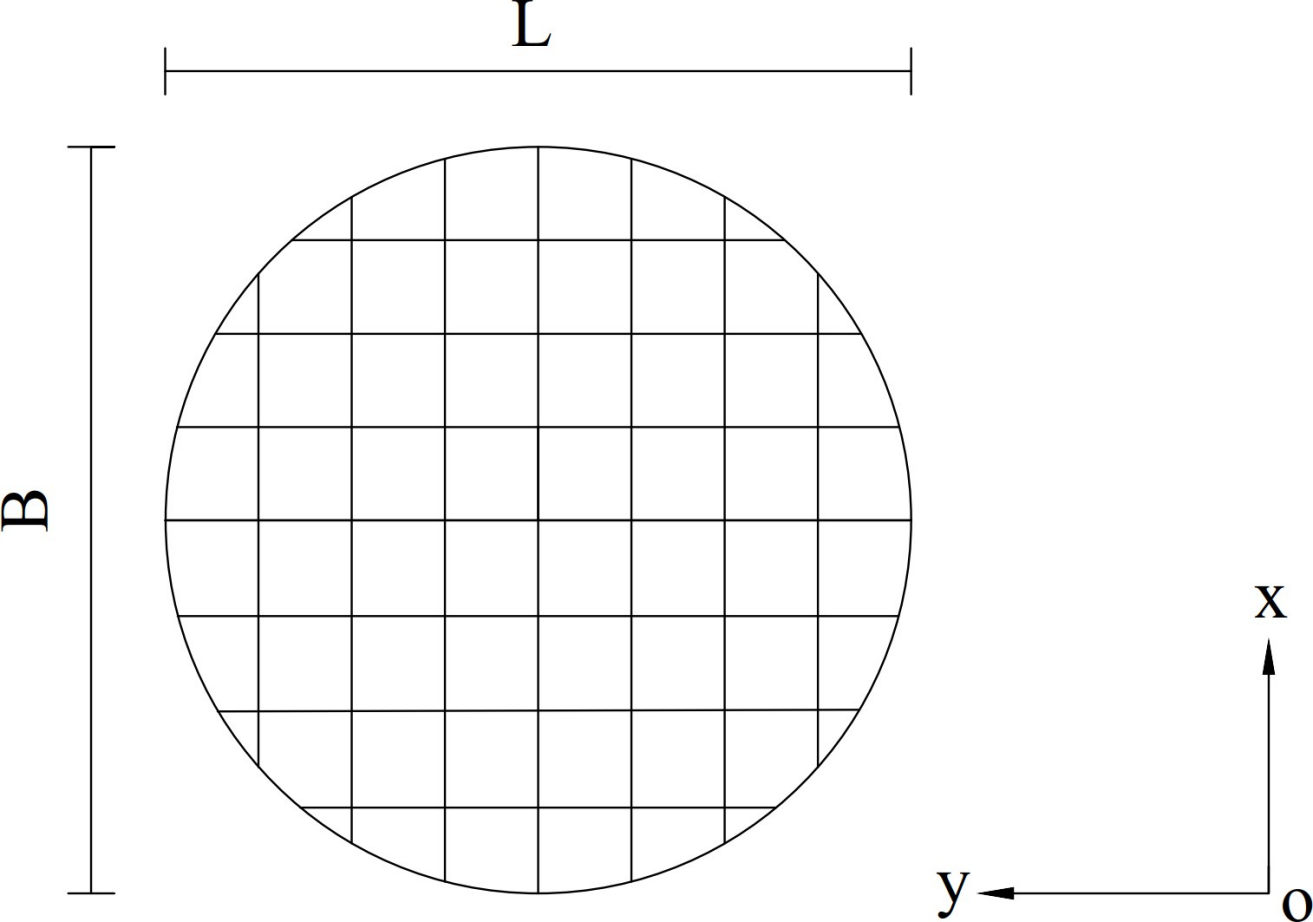
A plane view of the slip surface.

Example 2 is based on the dataset provided by Lu and Zhu [27]. Fig 10 schematically illustrates that a homogenous slope with a height of *H* = 40*m*, and strength parameters of soil *c*′ = 30 *kPa*, *φ*′ = 30 *kPa*, and *γ* = 22 *kN*/*m*^3^. The slip surface is an ellipsoid described by the equation:

$$\frac{{\left(x-40\right)}^{2}}{40^{2}}+\frac{y^{2}}{0.5L^{2}}+\frac{{\left(z-40\right)}^{2}}{40^{2}}=1$$ (25)

where B is the length of the slip surface in x direction (Fig 11). For L = 1,2,3,6 and 8 times the height of the slope, the proposed method’s safety factor *F*_*s*_ is calculated by the summaries in Table 3. This example intends to discuss the effect of the length of slip surface on the safety factor and compare the effect to that of different three-dimensional methods.

**Table 3:** Calculated results of slope stability by different three-dimensional limit equilibrium method for example 2.

Methods	L/H				
	1	2	3	6	8
1. Xing [12]	1.37	1.26	1.23	1.22	1.21
2. Hungr et al. [13]	1.34	1.24	1.22	1.21	1.21
3. Chen and Zhu [17]	1.25	1.19	1.18	1.17	1.17
4. Hovland [18]	1.15	1.14	1.14	1.15	1.15
5. Lu and Zhu [27]	1.29	1.21	1.19	1.18	1.17
6. Zhu et al. [28]	1.21	1.20	1.19	1.18	1.17
7. Present study	1.22	1.20	1.19	1.18	1.17

**Fig 12:**
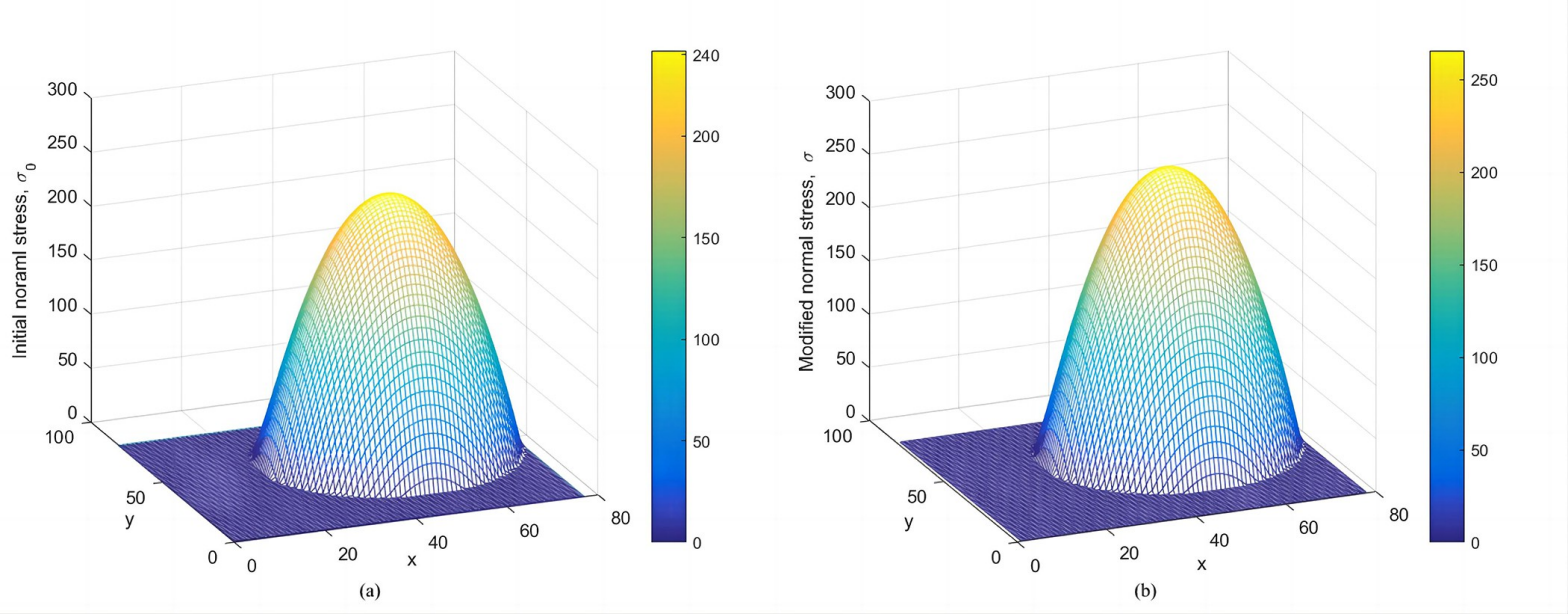
The distribution of normal stress for example 2.

The trend of the safety factor becomes more stable as the L/H ratio increases, and it shows good agreement with all methods from Table 3. Fig 12 indicates that the distribution of the modified normal stress over the slip surface is positive. By confirming that the value of the modified normal stress is non-negative, these results verify that the current method is reliable and acceptable.

### 3.3. Example 3: A circular slip surface adapted from Xing [12]

**Fig 13:**
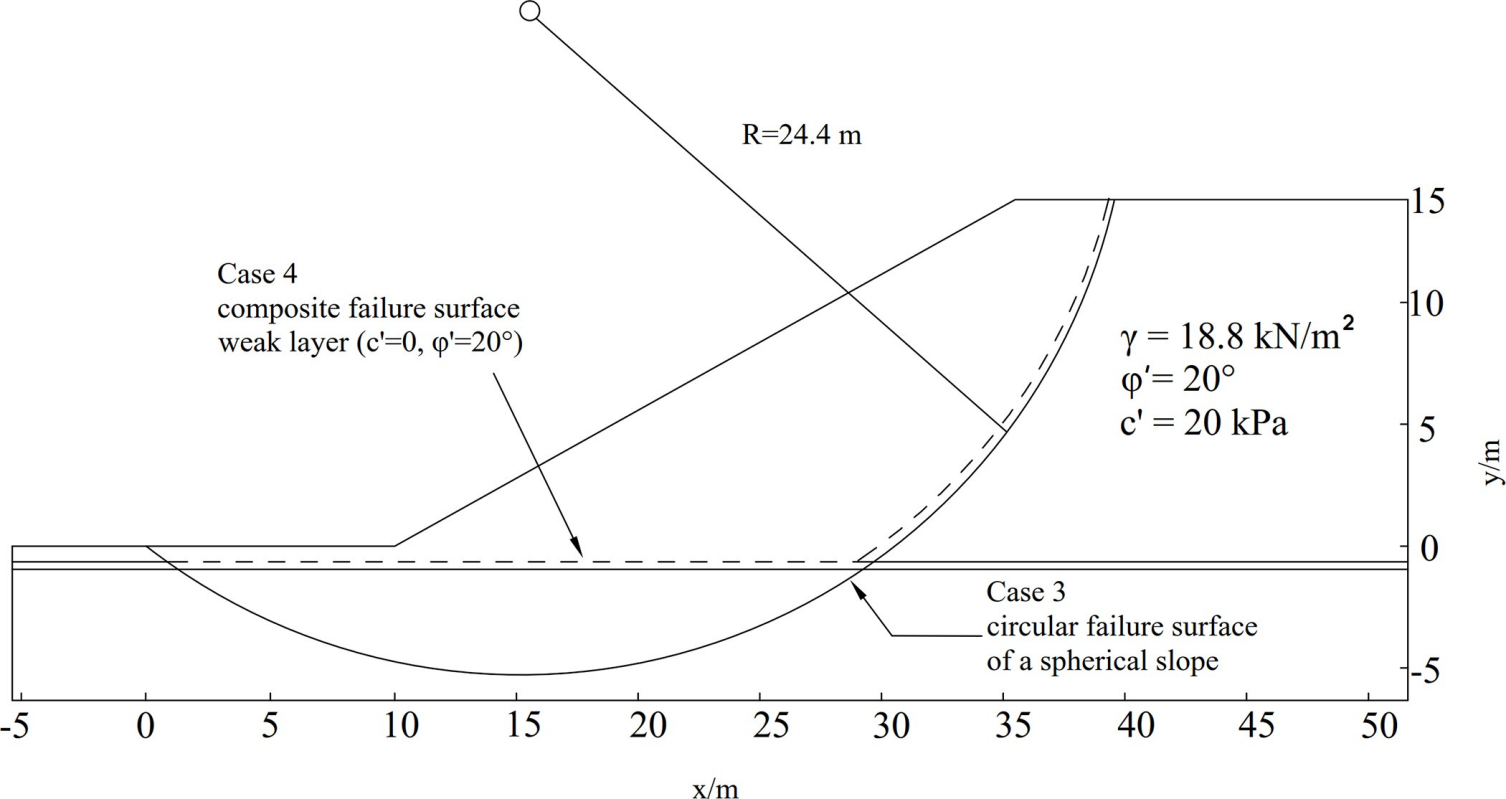
Cross-section and parameters of a spherical slope.

The slope height *H* = 12*m* and the slope ratio is 1:2; There are two cases in this example, (1) Case 3 with cohesion of *c*′ = 20*kPa*, internal angle of *φ*′ = 20°, and unit weight *γ* = 18.8 *kN*/*m*^3^, and (ii) Case 4 with *c*′ = 0, and *φ*′ = 20°. The cross-section and parameters of the spherical slope are shown in Fig 13. The slip surface can be numerically described as:

$${\left(x-15.1\right)}^{2}+y^{2}+{\left(z-19.1\right)}^{2}=24^{2}$$ (26)

Table 4 presents a comparison of calculated Fs values using different methods. The discrepancies between the current method and that of Liu [32] are about 3.57% and 1.47% for Case 3 and 4, respectively. This discrepancy is due to Liu [32] employing a rigorous limit equilibrium method, which yields relatively conservative results. The proposed method, when compared to the method of Zhu et al. [28], shows only minor differences of 0.08% and 0.06% for Cases 3 and 4, respectively. This demonstrates the proposed method’s effectiveness in analyzing slope stability for the circular slip surface, as indicated by the minimal error rates. As shown in Table 5, *K*_*c*_ decreases as *F*_*s*_ increases, in both Cases 3 and 4. Reliable *F*_*s*_ is determined when *K*_*c*_ approaches to zero.

**Table 4:** Comparison of calculated *F*_*s*_ values by different methods.

Methods	Case 3		Case 4	
	*F*_*s*_	Error	*F*_*s*_	Error
The proposed method	2.382	-	1.561	-
Zhu and Qian [26]	2.385	0.13%	1.567	0.38%
Zhu et al.[28]	2.380	0.08%	1.560	0.06%
Liu [32]	2.297	3.57%	1.538	1.47%

Note: The error refers to the difference between other methods and the proposed method.

**Table 5:** Variations of *F*_*s*_ and *K*_*c*_ values.

Iteration Step	Case 3		Case 4	
	*F*_*s*_	*K*_*c*_	*F*_*s*_	*K*_*c*_
1	1	2.10580	1	1.0288
2	0.9	2.49940	0.9	1.3287
3	1.535	0.85950	1.3431	0.3089
4	1.868	0.43350	1.4773	0.1094
5	2.207	0.12640	1.5509	0.0131
6	2.346	0.02460	1.5609	0.0006
7	2.379	0.00170	1.5614	0.000003
8	2.382	0.00002	-	-

**Fig 14:**
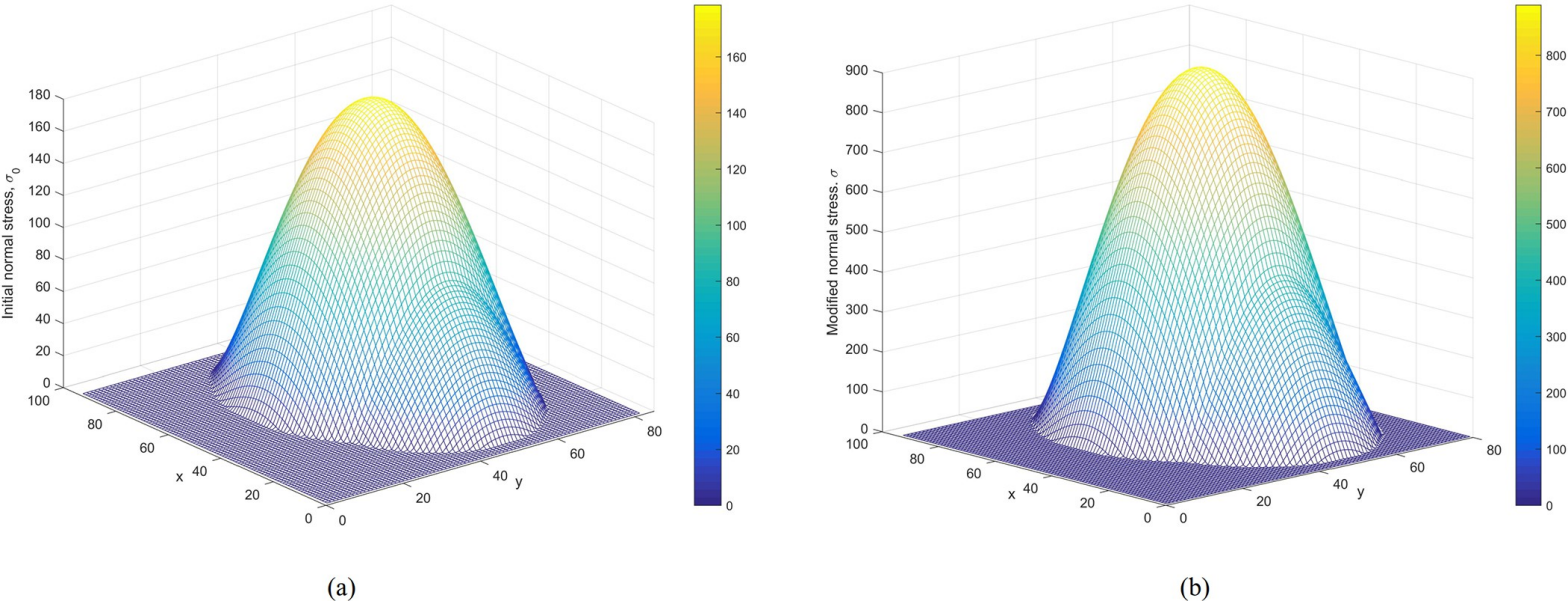
Case 3: (a) The distribution of the initial normal stress over the circular slip surface (b) The modified normal stress over the circular slip surface.

**Fig 15:**
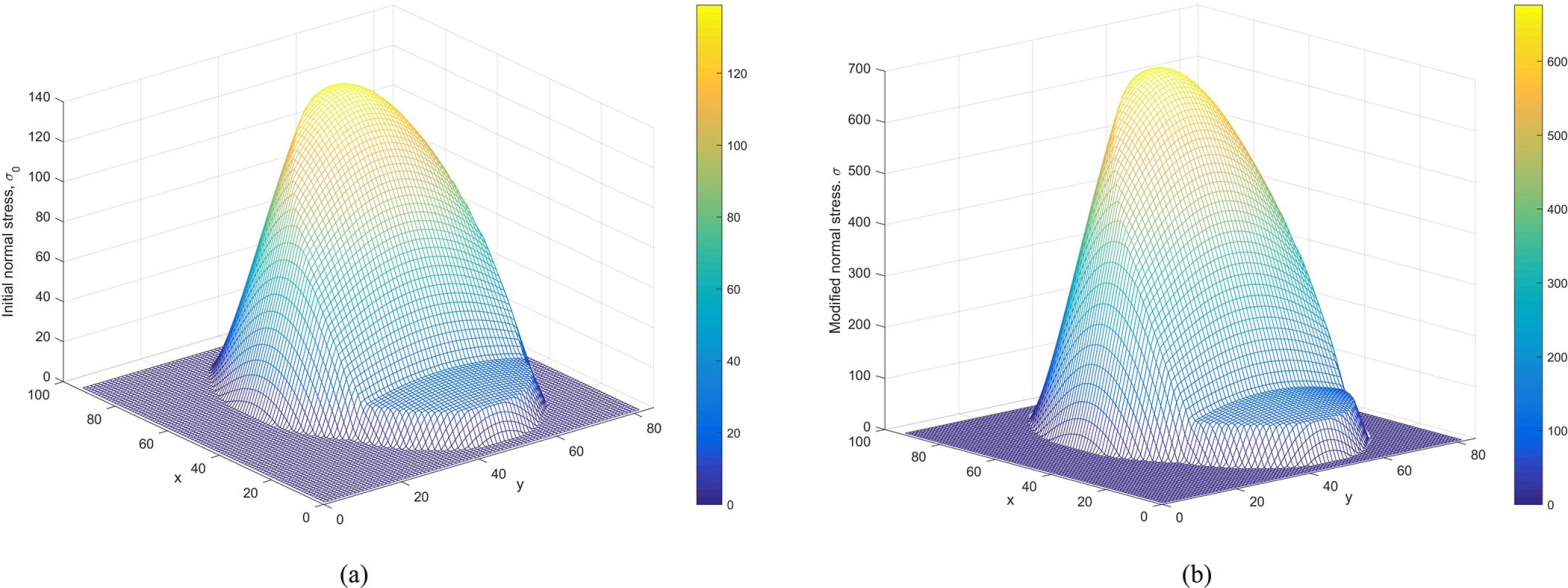
Case 4: (a) The distribution of the initial normal stress over the composite slip surface. (b) The distribution of the modified normal stress over the composite slip surface.

Figs 14(A) and 15(A) represent the initial normal stress over the slip surface for Cases 3 and 4. As shown in Figs 14(B) and 15(B), distributions of modified normal stresses in Cases 3 and 4 are smooth and positive, indicating acceptable results.

### 3.4. Example 4: Wedge failure adapted by Hoek and Bray [33]

**Fig 16:**
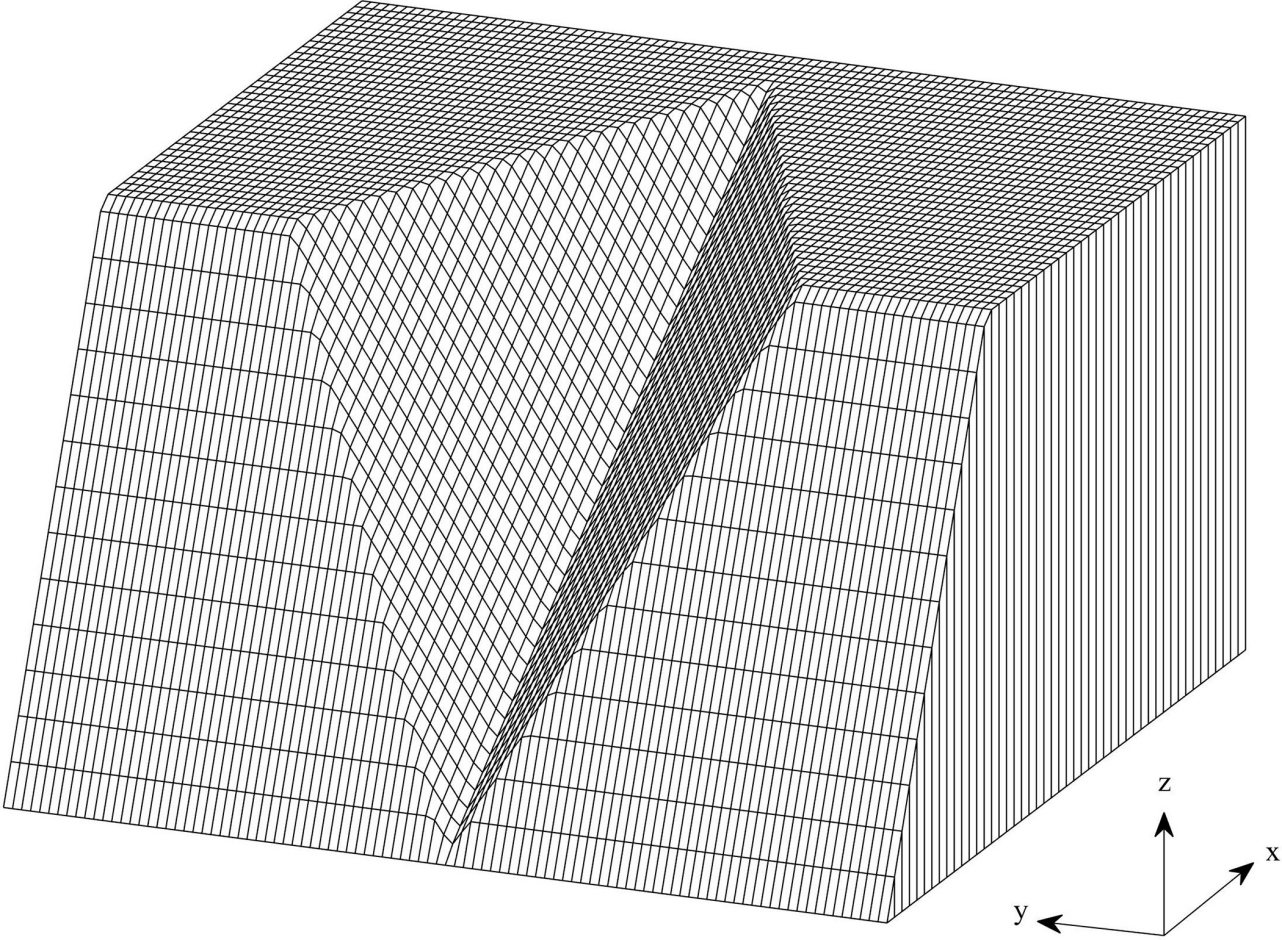
The 3D slope profiles for symmetric wedge slip surface.

**Fig 17:**
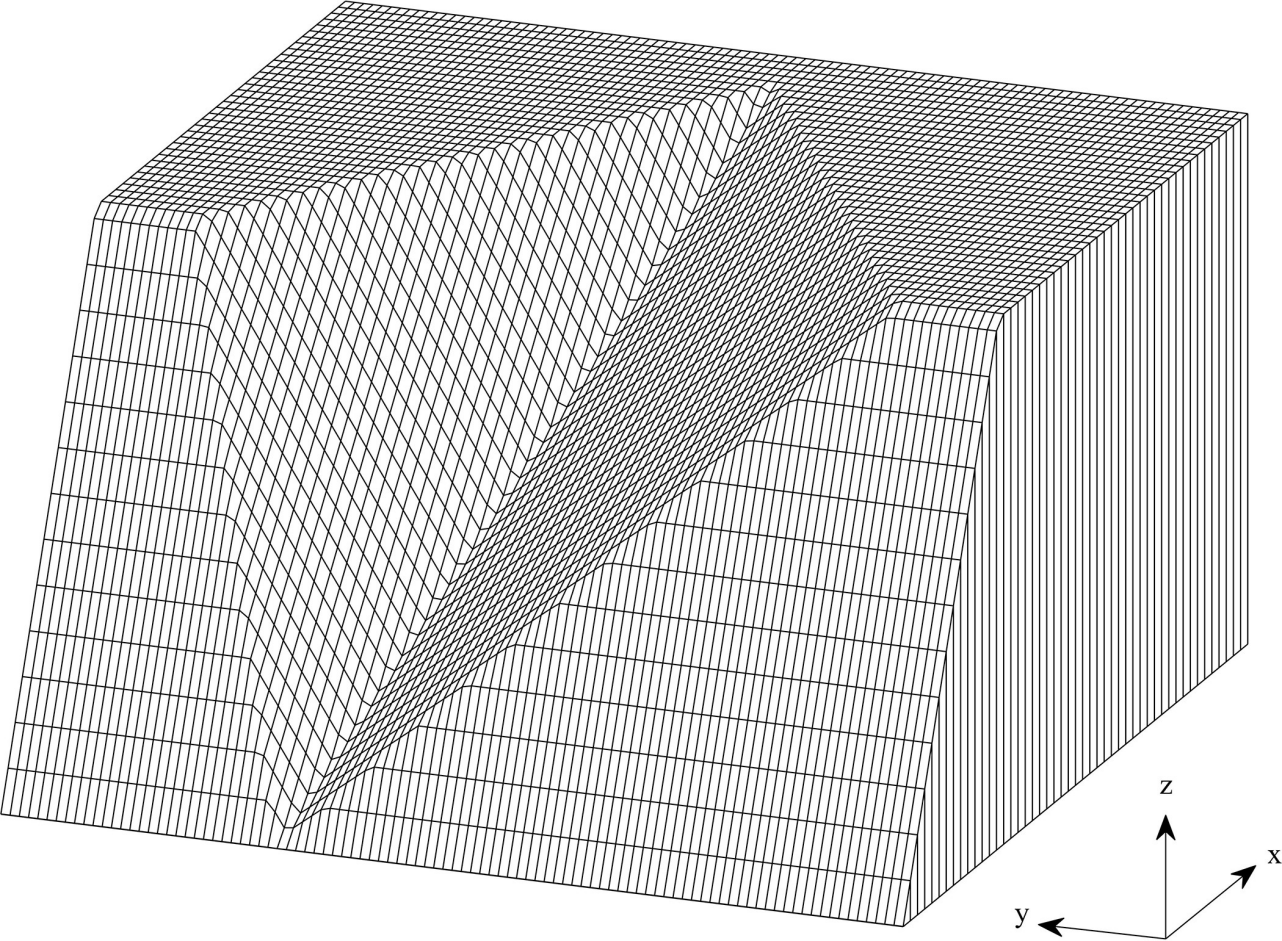
The 3D slope profiles for an asymmetric wedge slip surface.

The stability of wedge in rock mechanics is a typical issue concerning three-dimensional limit equilibrium. The calculated examples involve both symmetric and asymmetric geometric wedge shapes. To test the validity of the proposed method, the results obtained by the proposed method are compared with those by Zhu et al. [28], Guo et al. [34], Jiang and Zhou [35], and Su and Shao [36], respectively. The geometric shape diagram for symmetric and asymmetric wedge in example 4 are shown in Figs 16 and 17. In these two examples, left and right failure planes of wedges take the same shear strength. As to the symmetric wedge, *c*′ = 0.02*MPa* and *φ*′ = 20°, while as to the asymmetric wedge, *c*′ = 0.05*MPa*, and *φ*′ = 30°. The rock unit weight is 26 kN/m^3^. The parameter and geometry information using in the method are listed in Table 6.

**Table 6:** Information of discontinuity planes forming the wedges.

Wedge type	Surface	Dip (°)	Dip direction(°)	Friction angle (°)	Cohesion (kPa)	Height of wedge (m)
Symmetric	Left failure plane	45	115	20	20	-
	Right surface	45	245	20	20	-
	Slope surface	60	180	-	-	100
	Top surface	10	180	-	-	100
Asymmetric	Left failure plane	40	120	30	50	-
	Right failure plane	60	240	30	50	-
	Slope surface	60	180	-	-	100
	Top surface	0	180	-	-	100

Note: The rock unit weight = 26 kN/m^3^.

As shown in Table 7, there exist minor (and practically negligible) differences between the values calculated using the proposed method and those calculated by other methods. These discrepancies could be due to the imperfect replication of slip surface geometry. For an asymmetric wedge, Su and Shao [36] present significantly different results, as they take into account the effect of Poisson’s ratio.

**Table 7:** A comparison of the safety factors for the wedge solution.

Methods	The factor of safety, *F*_*s*_	
	Symmetric	Asymmetric
Zhu et al. [28]	1.280	1.644
Hoke and Bray [33]	1.293	1.640
Guo et al. [34]	1.337	1.653
Jiang and Zhou [35]	1.275	1.629
Su and Shao [36]	1.294	1.491
The proposed method	1.280	1.645

**Fig 18:**
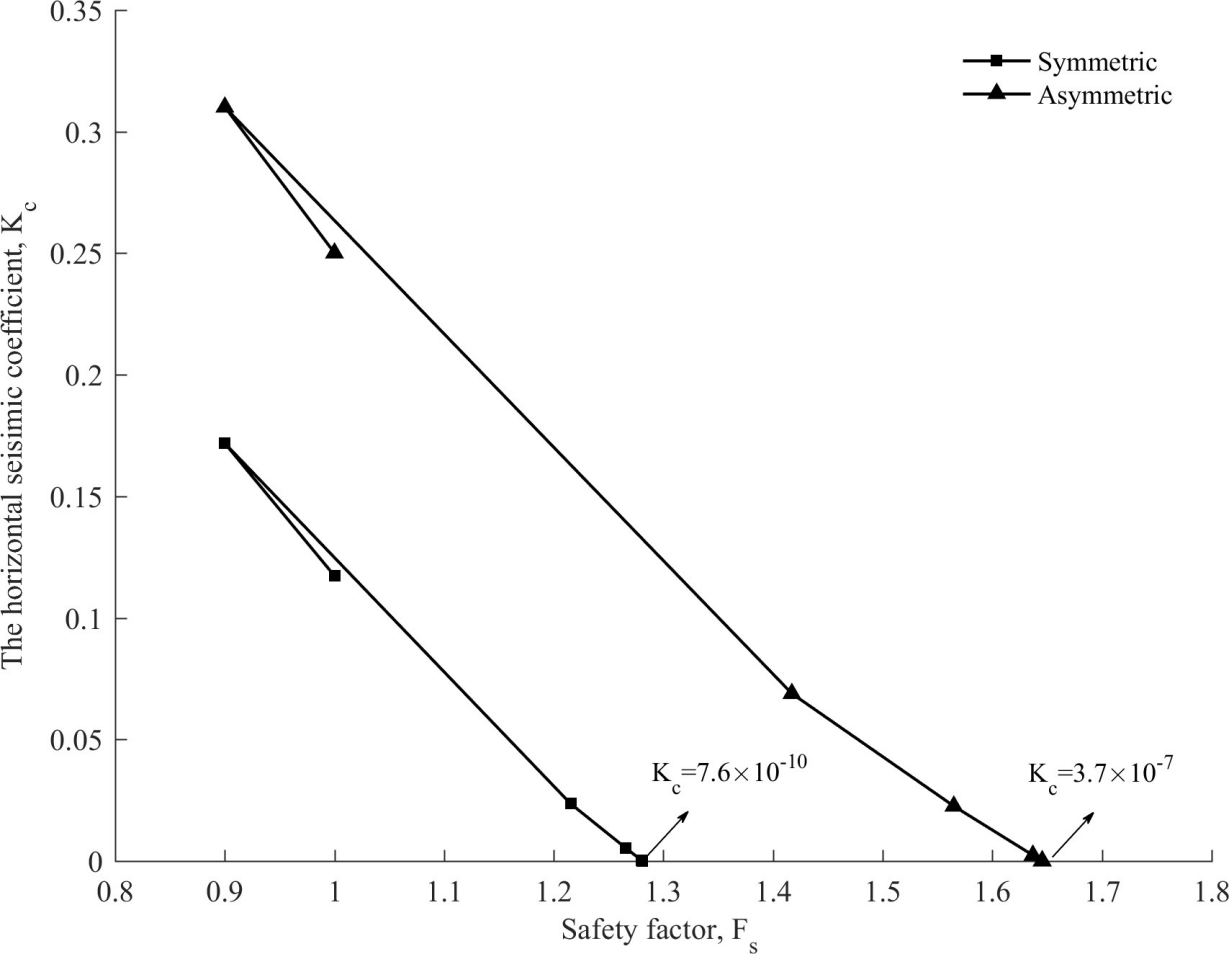
Correlations between *K*_*c*_ and *F*_*s*_ under different semiaxis ratios for symmetric and asymmetric slip surfaces.

A strong correlation exists between *K*_*c*_ and *F*_*s*_, as shown in Fig 18. *F*_*s*_ = 1 is used as the initial safety factor for six steps of iteration. This case used 1 to start iteration of the initial safety factor, and ended the program through six steps. The values of instantaneous *K*_*c*_ decrease with the increase of *F*_*s*_ values at step 2 and keep decreasing until the safety factor is stable. This phenomenon is a result of the fact that the horizontal seismic coefficient has a great influence on the safety factor.

**Fig 19:**
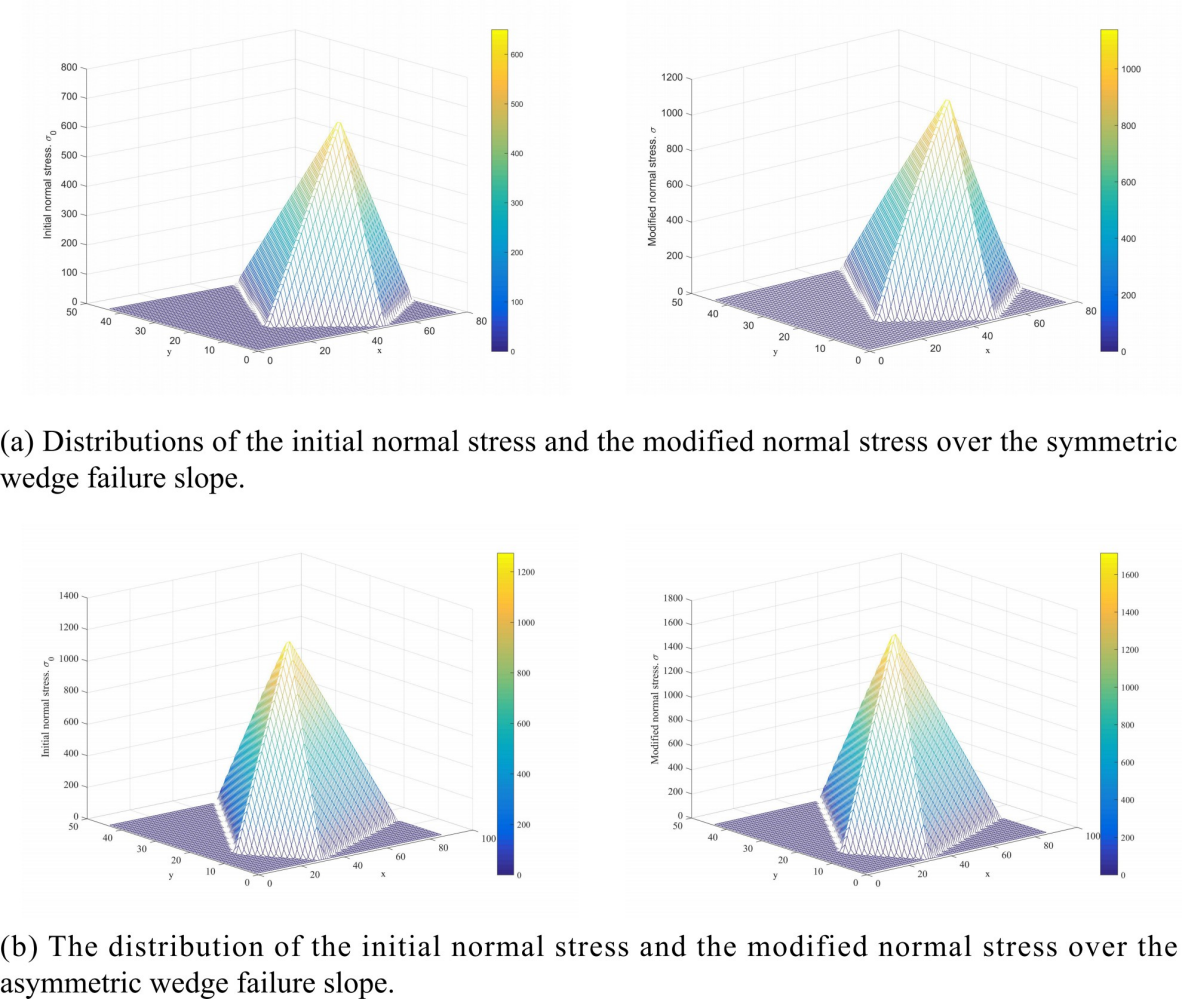
Comparison between the distribution of the initial normal stress and that of the modified normal stress for (a) symmetric and (b) asymmetric failure slopes.

Fig 19 displays a comparison between the distribution of the initial normal stress and the distribution of the modified normal stress for asymmetry and symmetry wedge failure. The modified normal stress sketches show that the modified normal stress is non-negative, verifying the rationality of the results.

### 3.5. Discussion of the advantages

This paper eliminated two moment balance equations considered relatively minor in engineering to simplify the problem-solving process. Only three force equilibrium equations in the (x, y, z) directions and a one-moment equilibrium equation in the (z) direction were considered. This method streamlines the calculation process without compromising computational accuracy.

Compared with the rigorous 3-D method, this method simplified the formulas of computation and avoided the negative normal stress at the edges of the slip surface. We found that disregarding the two secondary moment equilibrium conditions does not affect the accuracy of the results. It points out that the error of Fs is within the acceptable level. As shown in Table 8, compared to the rigorous method, the error with Case 1 is 0.04%, with Case 2 is 0.06%, with Case 3 is 0.08%, and with Case 4 is 0.06%, respectively. The error can be negligible. The calculation process of this method is easy to program, which serves to decrease the iteration and computation times. From Table 8, the iteration times are decreasing in all Cases. This indicates an improvement in computational efficiency.

**Table 8:** Compare the results between rigorous 3-D method and simplified method in iteration times and the safety factor.

Method	Case 1		Case 2		Case 3		Case 4	
	Times	*F*_*s*_	Times	*F*_*s*_	Times	*F*_*s*_	Times	*F*_*s*_
Rigorous 3-D method [28]	8	2.141	7	1.661	9	2.380	8	1.560
Proposed method	6	2.142	5	1.662	7	2.382	6	1.561
Error	-	0.04%	-	0.06%	-	0.08%	-	0.06%

## 4. Conclusion

1. This proposed method is a simplified three-dimensional method based on the limit equilibrium method and the method of modifying normal stress distribution over the slip surface. The novelty of this method lies in incorporating the horizontal seismic coefficient as an intermediate variable; it employed a "clever trick" of introducing the horizontal seismic force coefficient to linearize the equations. Such a method involves force equilibrium in three directions and moment equilibrium in the vertical direction. The safety factor of slope can be determined by finding the minimum value of horizontal seismic coefficient. The smooth and positive distribution of the modified normal stress verifies the effectiveness of the proposed method and the reliability of the obtained results.
2. Meaningful comparisons were made between symmetric and asymmetric problems to investigate the influence of the horizontal seismic coefficient on the factor of safety. The slope safety factor tends to stabilize as the horizontal seismic coefficient decreases. From an engineering perspective, this method eliminates two secondary moments equilibrium conditions without compromising calculation accuracy and computational efficiency. It maintains calculation accuracy and achieves good agreement with a rigorous 3-D method by utilizing four equilibrium equations to solve the problem.
3. Most of the traditional methods for determining the three-dimensional limit equilibrium of the slopes are associated with complex mathematical deviations and tedious calculations. However, the proposed method is simple in calculation, fast in convergence, wide in application, and easy to program. Although it needs iterations, a few steps are enough to produce convergent results.
